# Characterization of nucleolar localization signals in the avian infectious bronchitis virus nucleocapsid protein and their critical role in viral replication

**DOI:** 10.1128/jvi.00433-26

**Published:** 2026-06-04

**Authors:** Liwei Zhang, Yingfei Li, Jun Zhao, Rong Liang, Jing Zhao, Ye Zhao, Guozhong Zhang

**Affiliations:** 1 State Key Laboratory of Veterinary Public Health and Safety, College of Veterinary Medicine, China Agricultural University 630101 , Beijing, People’s Republic of China; 2 Key Laboratory of Animal Epidemiology of the Ministry of Agriculture, College of Veterinary Medicine, China Agricultural University 630101 , Beijing, People’s Republic of China; University of Kentucky College of Medicine, Lexington, Kentucky, USA

**Keywords:** infectious bronchitis virus, nucleocapsid protein, nucleolar localization signal, viral replication

## Abstract

The nucleocapsid (N) protein of infectious bronchitis virus (IBV) is the most abundant viral protein in infected cells and is essential for virion assembly and for modulating host cellular processes. Although previous studies have reported nucleolar accumulation of IBV N, a comprehensive mapping of its nucleolar localization signals (NoLSs) is lacking. Here, we systematically mapped functional NoLSs within the IBV N protein and evaluated their contributions to viral replication. Under both infection and transient-transfection conditions, the N protein exhibited a dual cytoplasmic–nucleolar distribution. Analysis of EGFP-tagged N protein truncation mutants identified four candidate NoLSs (NoLS1–4) and a nuclear localization signal (NLS). Alanine-scanning mutagenesis established that NoLS3 (amino acids [aa] 330–349) and NoLS4 (aa 356–368) were the principal determinants of nucleolar targeting within the full-length protein, with this activity depending on basic residue clusters 2 and 3 in NoLS3 and cluster 1 in NoLS4. Recombinant virus rescue assays demonstrated that the double mutant strain (rYN-N-K356A/R359A) abolished nucleolar accumulation of N protein and showed significantly decreased replication levels at 24 and 36 h post-infection. Collectively, this study identifies specific basic residues in NoLS3 and NoLS4 as the molecular basis for IBV N nucleolar localization and demonstrates that nucleolar targeting is required for optimal IBV replication.

**IMPORTANCE:**

Avian infectious bronchitis virus (IBV) is a highly contagious pathogen that causes severe economic losses to the global poultry industry. While the N protein is known to localize to the nucleolus, the precise sequences of its NoLSs and their impact on viral replication, remain unclear. This study systematically identified the functional NoLSs of the IBV N protein and the essential basic residues within them. Crucially, we demonstrated that the nucleolar localization of the N protein plays an important role in viral replication. Furthermore, elucidating the specific motifs required for N protein functionality may inform the rational design of live-attenuated vaccines or antiviral approaches against IBV.

## INTRODUCTION

Infectious bronchitis virus (IBV), a gammacoronavirus, causes a highly contagious disease in chickens that inflicts substantial economic losses on the global poultry industry (1). As an enveloped, positive-sense single-stranded RNA [(+) ssRNA] virus, the IBV genome encodes four major structural proteins: spike (S), envelope (E), membrane (M), and nucleocapsid (N) (2). Among these, the N protein is the most abundant and highly conserved viral protein during infection of host cells (3). The N protein is a basic phosphoprotein with 409 amino acids (~45–50 kDa), consisting of an N-terminal domain (NTD) that primarily binds RNA and a C-terminal domain (CTD) that mediates dimerization and contributes to higher-order oligomerization; in addition, the C-terminal region contains a functional nuclear export signal (NES) (4–6). It mediates encapsidation of the viral genome, forming a helical ribonucleoprotein (RNP) complex and thereby protecting the viral RNA from degradation (4, 7). Beyond its fundamental structural role, the N protein modulates multiple host-cell processes, including the inhibition of host protein synthesis, delay of cell-cycle progression, and antagonism of innate immune responses (8–10).

The nucleolus is the nuclear subcompartment primarily responsible for ribosome biogenesis (11). Structurally, it is partitioned into three distinct subcompartments: the fibrillar center (FC), the dense fibrillar component (DFC), and the granular component (GC), which are primarily responsible for ribosomal DNA (rDNA) transcription, early processing of pre-ribosomal RNA (pre-rRNA), and late-stage ribosomal subunit assembly, respectively (12). Beyond its canonical function as a ribosome factory, the nucleolus is increasingly recognized as a dynamic regulatory center involved in cellular stress sensing, protein sequestration and post-translational modification, cell-cycle surveillance, and the DNA damage response (13–16). Although coronaviruses replicate and assemble mainly in the cytoplasm, multiple studies have reported nucleolar accumulation of the N protein from various coronaviruses, including IBV, severe acute respiratory syndrome coronavirus (SARS-CoV), porcine epidemic diarrhea virus (PEDV), transmissible gastroenteritis virus (TGEV), and mouse hepatitis virus (MHV), during infection or ectopic expression (17–20). The N protein colocalizes with and physically interacts with nucleolar marker proteins (21, 22). Nucleolar localization of the viral protein has also been observed in a variety of other enveloped RNA viruses and is thought to promote viral replication either by perturbing host nucleolar functions (e.g., rRNA biogenesis, cell-cycle regulation, and stress responses) or by recruiting nucleolar proteins (23–25).

Nucleolar localization of viral proteins is commonly mediated by nucleolar localization signals (NoLSs) enriched in basic amino acid residues (26). Although candidate NoLSs have been reported in multiple coronaviruses, these motifs display little sequence or positional conservation across different viruses, precluding their reliable identification by simple homology-based alignments. For example, the NoLS of PEDV (an alphacoronavirus) N protein was identified in the N-terminal region (^71^SNWHFYYLGTGPHGDLRYRT^90^), whereas the N protein of SARS-CoV (a betacoronavirus) harbors nucleolar-targeting activity within a central region (aa 226–289) (18, 19). In contrast, the NoLS of porcine deltacoronavirus (PDCoV; a deltacoronavirus) N protein localizes to the C-terminal region (^295^PTKDKKPDKQ DQSAKPKQQKKPKK^318^) (27). For the IBV N protein, Reed et al. reported an eight-residue basic motif in the N terminus (^71^WRRQARFK^78^) and proposed it as the NoLS (28). Interestingly, our results demonstrated that mutation of all basic residues within residues 72–85 (RRQARYKPGKGGRK), which encompasses the previously proposed NoLS together with nearby basic residues, did not abolish the nucleolar accumulation of the full-length N protein. This strongly indicates that the motif reported earlier is not the sole determinant driving nucleolar localization and that additional, as-yet-unidentified signals contribute to nucleolar targeting. Importantly, many early NoLS mapping studies employed truncated fragments or overexpression systems bearing large fluorescent tags, approaches that may not faithfully reflect the functional importance of these motifs in the context of authentic viral infection (28–30).

This study systematically identified functional NoLSs within the N protein of IBV and assessed their contributions to viral replication. Using EGFP-tagged N protein truncations combined with alanine-scanning mutagenesis, we mapped two functional NoLSs (NoLS3 and NoLS4) and elucidated the critical requirement of specific basic residues within the full-length N protein. Crucially, by employing an IBV reverse genetics system, we rescued wild-type recombinant viruses and mutant viruses impaired in N nucleolar localization and demonstrated that the disruption of N protein nucleolar localization substantially attenuates viral replication. This study refines our understanding of the role of N protein nucleolar localization in IBV biology and provides a rationale for exploring NoLSs as potential targets for live-attenuated vaccine design or antiviral strategies.

## RESULTS

### IBV N protein localizes to the nucleolus during viral infection and transfection

**Fig 1:**
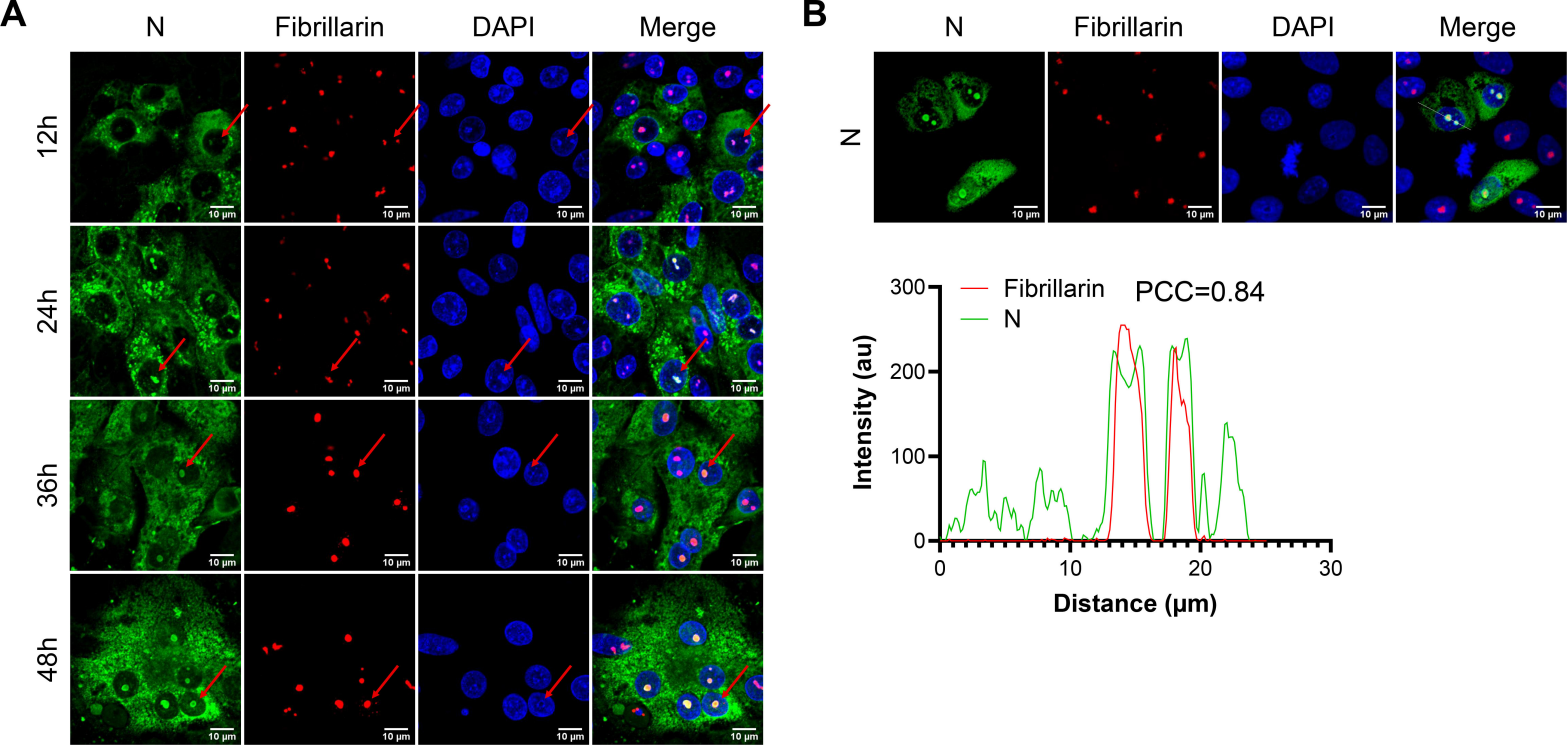
Nucleolar localization of IBV N protein during viral infection and transfection. (**A**) Subcellular localization of the IBV N protein during viral infection. CEK cells were infected with IBV at an MOI of 0.1, fixed at 12, 24, 36, and 48 hpi, and processed for IFA. Cells were immunolabeled with anti-IBV N and anti-fibrillarin antibodies, followed by incubation with secondary antibodies conjugated to Alexa Fluor 488 and Alexa Fluor 555. Nuclei were counterstained with DAPI. Red arrows indicate sites of colocalization between the IBV N protein and the nucleolar marker fibrillarin. Scale bars: 10 μm. (**B**) Subcellular localization of the N protein following plasmid transfection. Vero E6 cells were transfected with pCMV-Flag-N and fixed 24 h post-transfection. Samples were stained using the same IFA procedure as in panel A. Fluorescence intensity line-profiles and PCC were used to quantify the degree of colocalization between the N protein and fibrillarin. Scale bars: 10 μm.

To determine the subcellular localization of the IBV N protein, CEK cells were infected with the IBV YN strain at a multiplicity of infection (MOI) of 0.1 and analyzed via IFA at 12, 24, 36, and 48 hpi. Throughout infection, N localized predominantly to the cytoplasm but also accumulated within the nucleolus (Fig. 1A). To assess whether nucleolar targeting is an intrinsic property of N that occurs independently of other viral components, Vero E6 cells were transfected with a eukaryotic expression plasmid encoding full-length N. Twenty-four hours post-transfection (hpt), the subcellular distribution of ectopically expressed N closely mirrored that observed in infected CEK cells (Fig. 1B). Fluorescence intensity line-profile analysis demonstrated strong colocalization between N and the nucleolar marker fibrillarin (PCC = 0.84). These data confirm that the IBV N protein exhibits nucleolar localization in both infected and transfected cells.

### IBV N protein contains four NoLSs and one NLS

**Fig 2:**
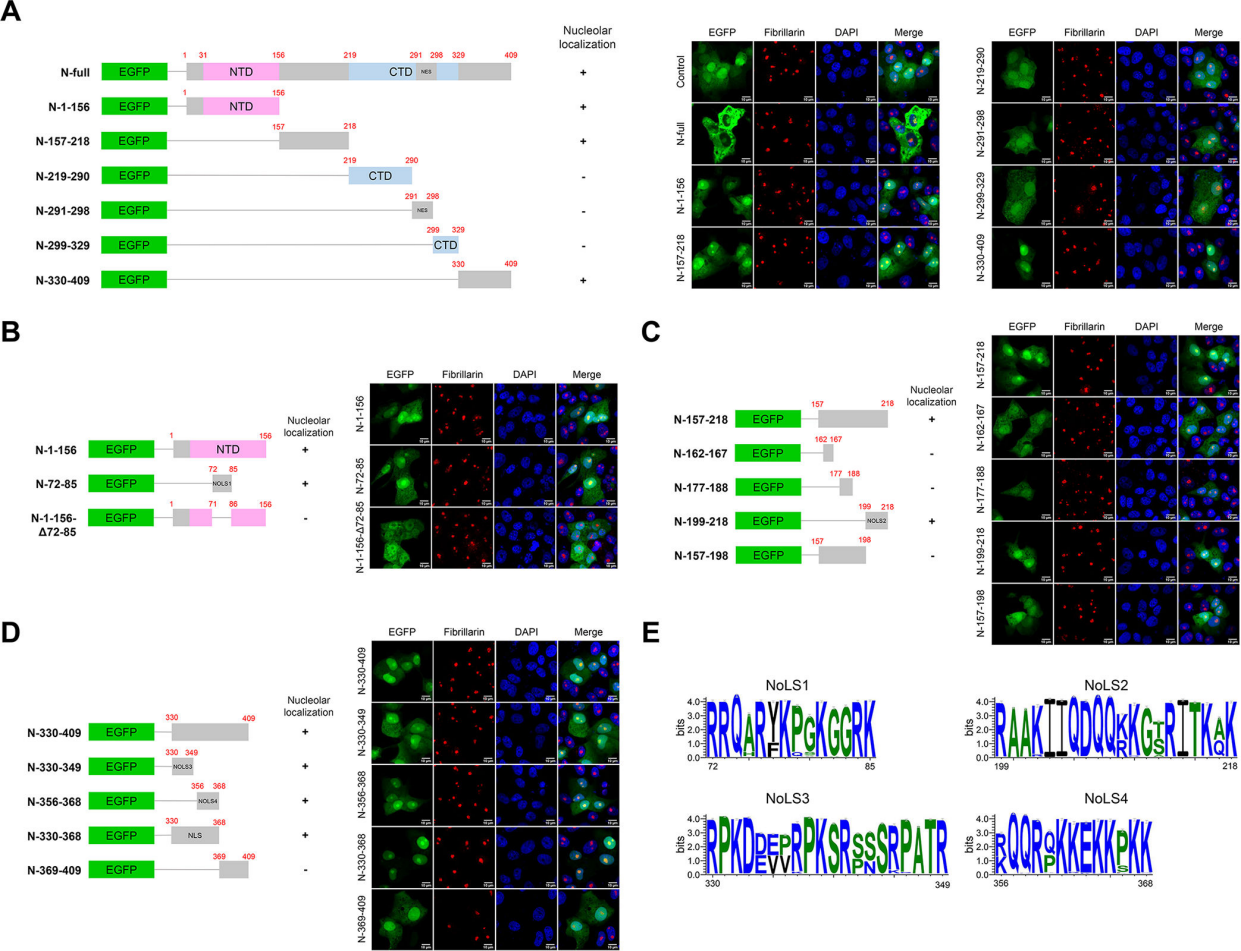
Mapping of four NoLSs and one NLS in the IBV N protein. (**A–D**) Nucleolar localization analysis of full-length IBV N protein and truncated mutants. Left panels present schematic representations of the full-length N protein and various truncated mutants fused to EGFP. Constructs were designed based on the structural domains of the N protein and regions rich in basic amino acids (aa 72–85, 162–167, 177–188, 199–218, 330–349, and 356–368). Right panels present representative confocal micrographs of the corresponding subcellular localization patterns. Vero E6 cells were individually transfected with the indicated expression plasmids. At 24 h post-transfection, the cells were fixed with 4% paraformaldehyde and processed for IFA. EGFP-fused proteins are shown in green, fibrillarin in red, and nuclei in blue. Scale bars: 10 μm. (**E**) Sequence conservation of the four identified NoLSs (NoLS1–NoLS4). Multiple sequence alignment of N proteins from diverse IBV strains was used to generate WebLogo representations. The height of each individual symbol represents the relative frequency of that specific amino acid at the corresponding position.

To identify regions of the IBV N protein responsible for nucleolar localization, we generated a series of EGFP-tagged N-protein truncation constructs based on its annotated domains (NTD, CTD, and NES). These constructs were transiently expressed in Vero E6 cells, and their subcellular localization was examined using confocal fluorescence microscopy. The initial screening revealed that similar to the full-length N protein, the truncation mutants N-1–156, N-157–218 and N-330–409 exhibited prominent nucleolar enrichment, whereas N-219–290, N-291–298, and N-299–329 displayed diffuse intracellular distribution and lacked specific nucleolar localization (Fig. 2A). Taken together, these results suggest that both the N-terminal region (aa 1–218) and the C-terminal region (aa 330–409) likely harbor multiple NoLSs. To precisely map the signal within aa 1–156, we focused on a short, basic motif spanning aa 72–85. This motif was sufficient, when fused to EGFP, to drive nucleolar accumulation; conversely, deletion of this segment from N-1–156 (N-1–156-Δ72–85) abolished nucleolar enrichment (Fig. 2B). Therefore, aa 72–85 were defined as the first NoLS (NoLS1). Similarly, truncation analysis of aa 157–218 revealed that nucleolar localization is entirely dependent on aa 199–218, which was designated NoLS2 (Fig. 2C). Fine mapping of the C-terminal region (aa 330–409) identified two independent nucleolar-targeting motifs: aa 330–349 (NoLS3) and aa 356–368 (NoLS4). Notably, the contiguous fragment encompassing both motifs (aa 330–368) displayed a pronounced dual nucleolar and nucleoplasmic localization pattern, suggesting that this combined region can function as an NLS. In contrast, aa 369–409 lacked nucleolar-targeting activity (Fig. 2D). Multiple sequence alignment of 784 IBV N-protein sequences retrieved from the NCBI database showed that all four NoLSs are enriched in basic residues (arginine [R] and lysine [K]) and are highly conserved across strains (Fig. 2E). In summary, we identified four NoLSs in the IBV N protein, namely NoLS1 (aa 72–85), NoLS2 (aa 199–218), NoLS3 (aa 330–349), and NoLS4 (aa 356–368), indicating that nucleolar localization of the N protein is likely mediated cooperatively by several dispersed, conserved basic motifs.

### NoLS3 and NoLS4 are the primary functional NoLSs of the IBV N protein

**Fig 3:**
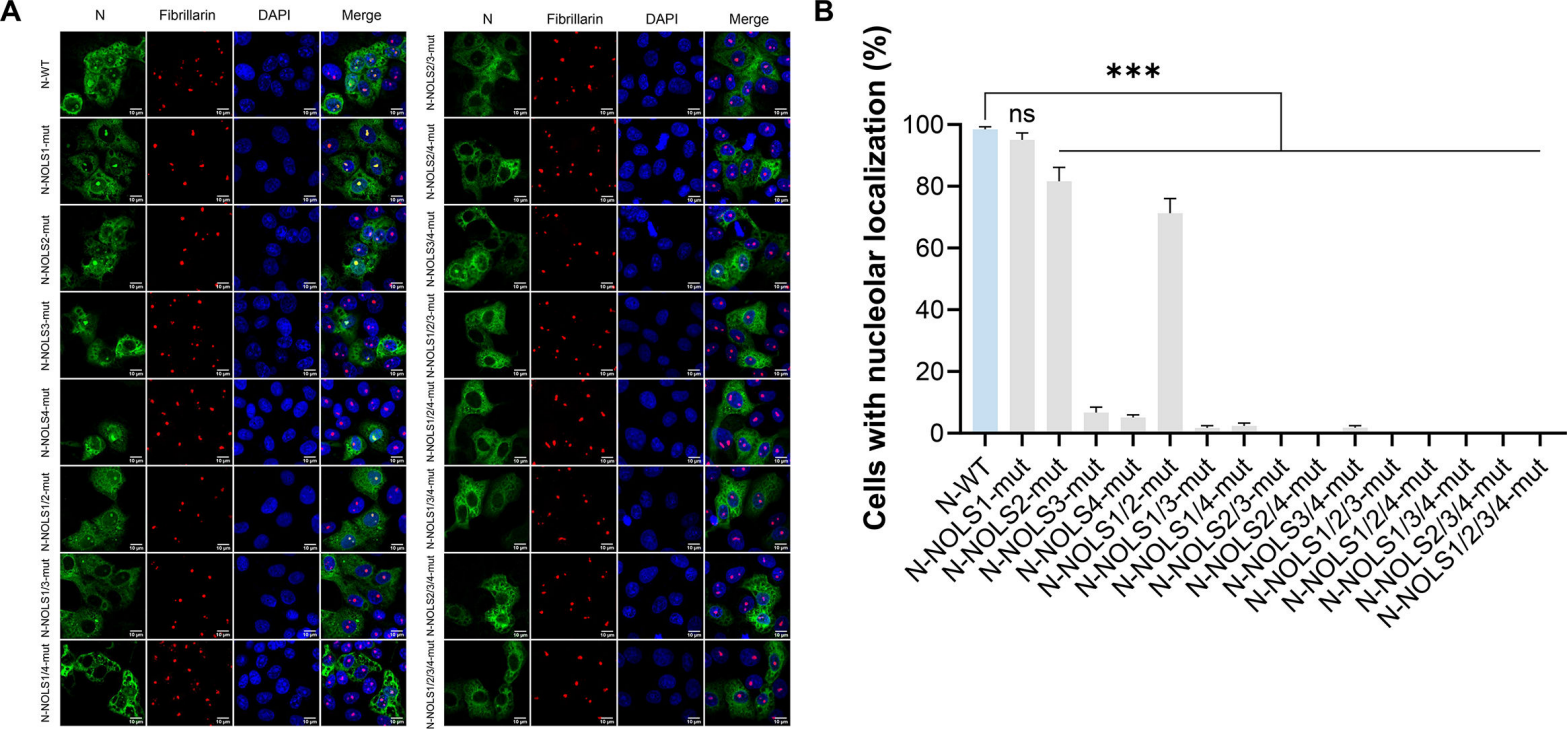
NoLS3 and NoLS4 are the primary functional NoLSs of the IBV N protein. (**A**) Subcellular localization of the wild-type and NoLS alanine substitution mutant N proteins. Vero E6 cells were individually transfected with plasmids expressing the wild-type N protein (N-WT), single-NoLS alanine-substitution mutants (all basic residues in the targeted NoLS replaced with alanine), or combinatorial NoLS mutants. At 24 h post-transfection, the cells were fixed and subjected to an IFA. N protein is shown in green, fibrillarin in red, and nuclei in blue. Scale bars: 10 μm. (**B**) Quantitative analysis of the N protein nucleolar localization. For each construct, the percentage of transfection-positive cells exhibiting distinct nucleolar accumulation of N protein was calculated. Data are shown as mean ± SEM from three independent experiments (*n* = 3); >200 transfected cells were scored in each experiment; ***P < 0.001; ns, not significant by one-way ANOVA, followed by Dunnett post hoc test.

To exclude the possibility that truncation constructs under overexpression conditions artificially expose basic regions and thereby alter intracellular localization, and to evaluate the functional contribution of the four previously identified NoLSs in the native conformation of the full-length N protein, we substituted all basic amino acids (R and K) within each signal region with alanine (A) and generated a series of single and combinatorial mutants. These constructs were expressed in Vero E6 cells and examined by confocal microscopy; nucleolar localization was quantified as the proportion of transfected cells exhibiting clear nucleolar accumulation of N (Fig. 3A and B). Individual or combined mutations in the N-terminal signals NoLS1 and NoLS2 (N-NoLS1-mut, N-NoLS2-mut, and N-NoLS1/2-mut) produced little change in nucleolar targeting, with levels of nucleolar localization comparable to wild-type N (N-WT). By contrast, any single or combined mutants that harbor alterations in the C-terminal signals NoLS3 or NoLS4 displayed a pronounced loss of nucleolar localization: fewer than 10% of cells showed nucleolar localization of N. Collectively, these data indicate that although NoLS1 and NoLS2 can contribute to nucleolar targeting in truncated fragments, the C-terminal NoLS3 and NoLS4 are the principal determinants of nucleolar localization in the context of the intact N protein, and efficient translocation to the nucleolus requires the cooperative integrity of both C-terminal NoLSs.

### Residues R337, K339, R341, R345, and R349 are critical for NoLS3 function

**Fig 4:**
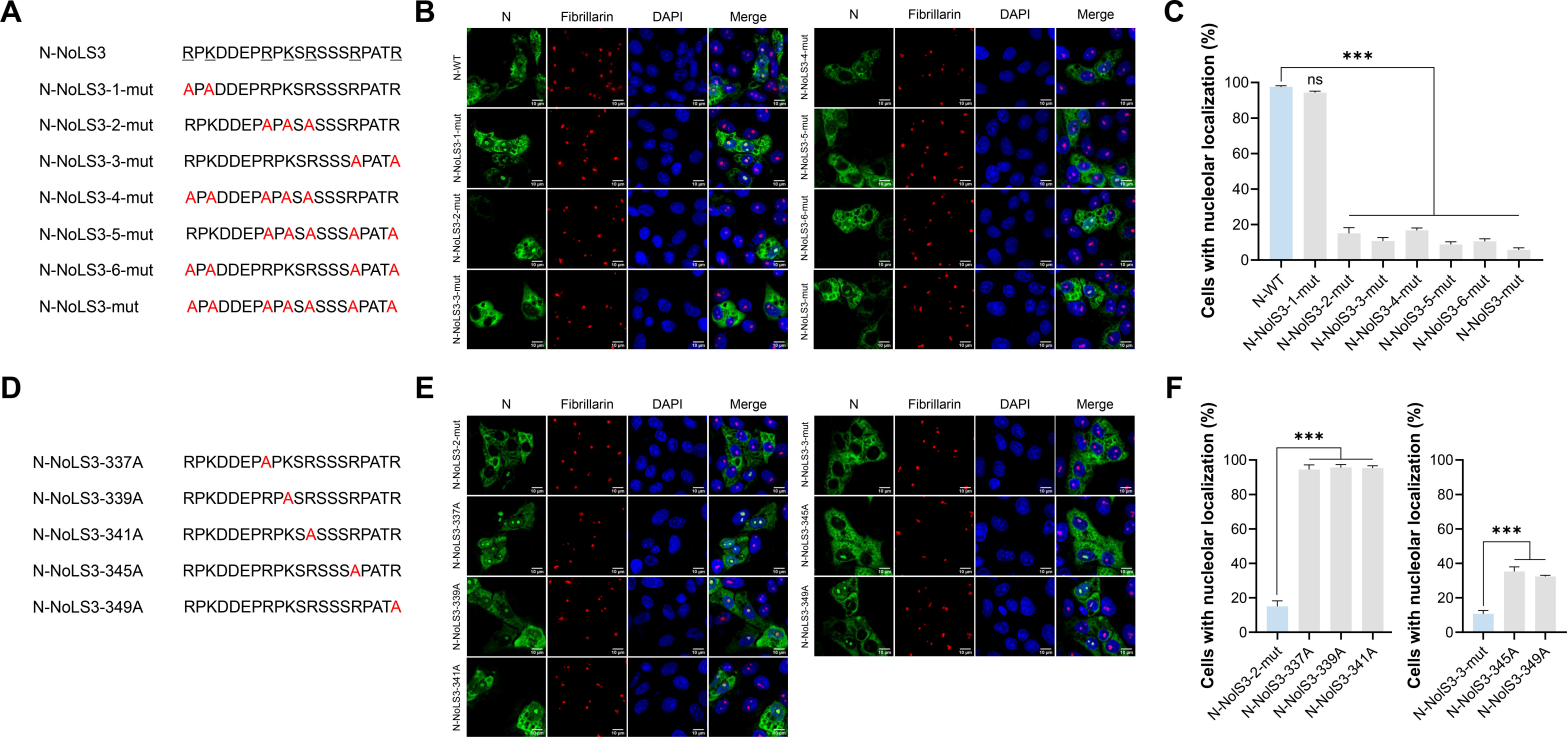
Cluster and single-point mutational analyses reveal critical basic residues for NoLS3 function. (**A**) Schematic representations of the amino acid sequences for the wild-type NoLS3 and its clustered alanine substitution mutants. The original basic amino acid residues are underlined, and the introduced alanine substitutions are indicated by red "A." (**B**) Vero E6 cells were individually transfected with plasmids expressing the indicated clustered mutants. At 24 h post-transfection, the cells were fixed and subjected to an IFA. N protein is shown in green, fibrillarin in red, and nuclei in blue. Scale bars: 10 μm. (**C**) Quantitative analysis of the N protein nucleolar localization. The percentage of N-expressing cells exhibiting distinct nucleolar localization of the N protein was calculated for each group. (**D**) Schematic representations of the NoLS3 mutants with single alanine substitutions of basic amino acids. (**E**) Confocal microscopy images showing the subcellular localization of the corresponding single-point mutants in Vero E6 cells at 24 h post-transfection. The staining strategy is identical to that described in panel B. Scale bars: 10 μm. (**F**) Quantitative analysis of the nucleolar localization for each single-point mutant. Data are shown as mean ± SEM from three independent experiments (*n* = 3); >200 transfected cells were scored in each experiment; ***P < 0.001; ns, not significant by one-way ANOVA followed by Dunnett post hoc test (C and F).

To identify the critical amino acid residues mediating nucleolar localization within the NoLS3 sequence, we adopted a cluster mutagenesis strategy based on the clustered distribution of its basic residues. Specifically, NoLS3 was divided into three clusters of basic residues: cluster 1 (^330^RPK^332^), cluster 2 (^337^RPKSR^341^), and cluster 3 (^345^RPATR^349^). All basic residues within each cluster were substituted with alanine to generate single-cluster and various combinatorial multi-cluster mutants (Fig. 4A). Confocal microscopy combined with quantitative analysis revealed that mutation of cluster 1 alone (N-NoLS3-1-mut) did not significantly affect nucleolar localization of the N protein compared with the wild type. By contrast, clustered mutations of cluster 2 (N-NoLS3-2-mut) or cluster 3 (N-NoLS3-3-mut) markedly reduced the frequency of nucleolar localization, and all multi-cluster mutants involving cluster 2 or cluster 3 (N-NoLS3-4-mut, N-NoLS3-5-mut, and N-NoLS3-6-mut) exhibited severely impaired nucleolar targeting (Fig. 4B and C). To further assess whether a specific single amino acid within clusters 2 and 3 is decisive for nucleolar targeting, a series of point mutants targeting individual basic residues was constructed (Fig. 4D). Although the combined mutation of cluster 2 (N-NoLS3-2-mut) produced a marked loss of nucleolar localization, any individual single-point substitution within this region retained nucleolar targeting of the N protein. In cluster 3, single substitutions at R345 or R349 (N-NoLS3-345A and N-NoLS3-349A) reduced nucleolar localization to ~30%, which nevertheless remained substantially higher than the level produced by mutation of the entire cluster 3 (11%) (Fig. 4E and F). These results indicate that NoLS3-mediated nucleolar localization depends on the overall positive-charge environment and cooperative interactions between residues in clusters 2 and 3 rather than on a single dominant basic residue. In particular, residues 337R, 339K, 341R, 345R, and 349R are critical for mediating NoLS3 nucleolar targeting.

### Residues K356 and R359 are critical for NoLS4 function

**Fig 5:**
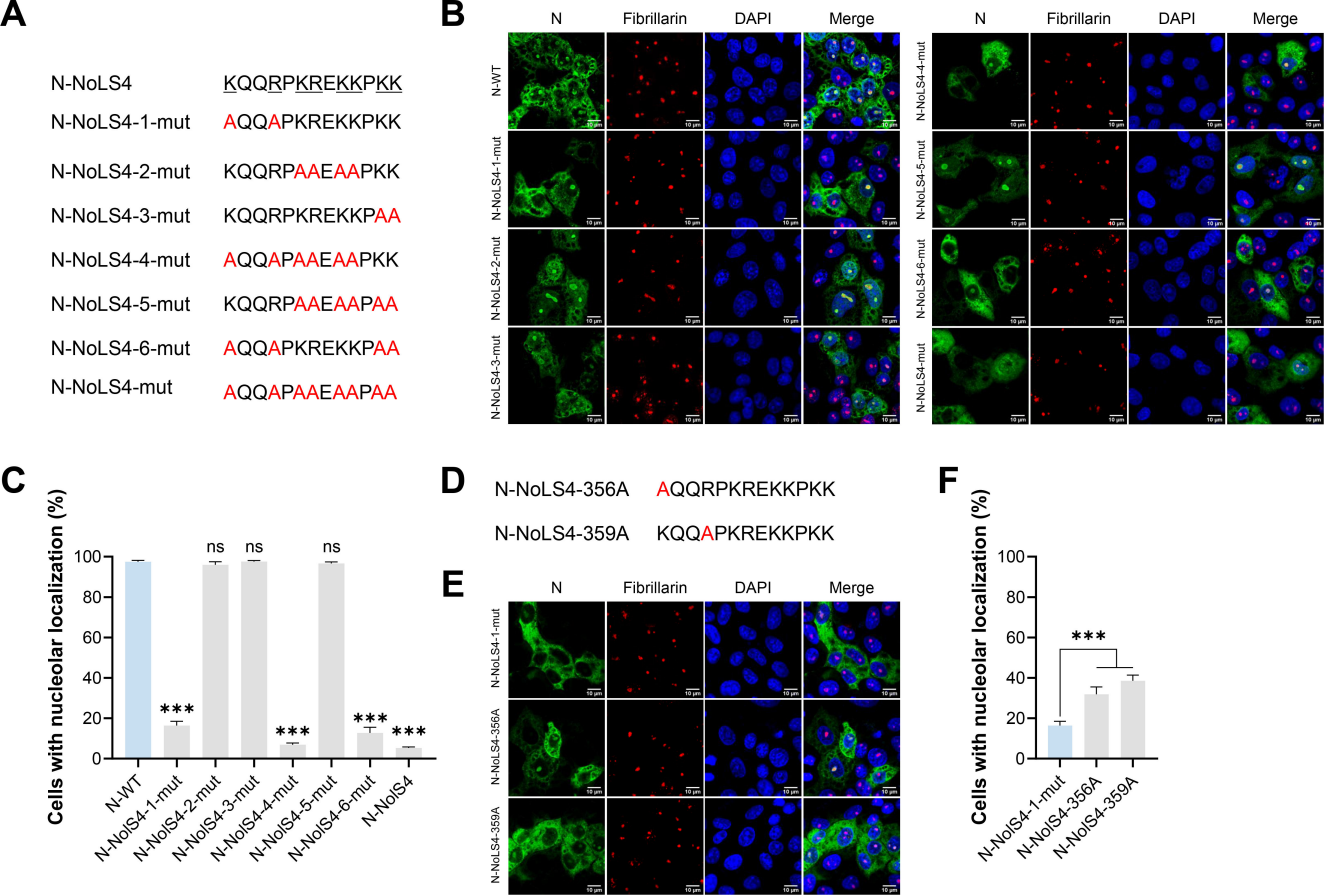
Cluster and single-point mutational analyses reveal critical basic residues for NoLS4 function. (**A**) Schematic representations of the amino acid sequences for the wild-type NoLS4 and its clustered alanine substitution mutants. The original basic amino acid residues are underlined, and the introduced alanine substitutions are indicated by red "A"s. (**B**) Vero E6 cells were individually transfected with plasmids expressing the indicated clustered mutants. At 24 h post-transfection, the cells were fixed and subjected to an IFA. N protein is shown in green, fibrillarin in red, and nuclei in blue. Scale bars: 10 μm. (**C**) Quantitative analysis of the N protein nucleolar localization. The percentage of N-expressing cells exhibiting distinct nucleolar localization of the N protein was calculated for each group. (**D**) Schematic representations of the NoLS4 mutants with single alanine substitutions of basic amino acids. (**E**) Confocal microscopy images showing the subcellular localization of the corresponding single-point mutants in Vero E6 cells at 24 h post-transfection. The staining strategy is identical to that described in panel B. Scale bars: 10 μm. (**F**) Quantitative analysis of the nucleolar localization for each single-point mutant. Data are shown as mean ± SEM from three independent experiments (*n* = 3); >200 transfected cells were scored in each experiment; ***P < 0.001 by one-way ANOVA followed by Dunnett post hoc test.

To identify the critical amino acids mediating nucleolar localization within NoLS4, we employed the aforementioned cluster mutation strategy. The basic amino acids in NoLS4 were divided into three clusters: cluster 1 (^356^KQQR^359^), cluster 2 (^361^KREKK^365^), and cluster 3 (^366^KK^368^), followed by the generation of corresponding single-cluster and multi-cluster mutants (Fig. 5A). Confocal microscopy coupled with quantitative analysis demonstrated that individual mutations in Cluster 2 (N-NoLS4-2-mut) or Cluster 3 (N-NoLS4-3-mut), or the simultaneous mutation of both regions (N-NoLS4-5-mut), did not substantially affect the nucleolar localization of the N protein. By contrast, any mutation involving Cluster 1 (N-NoLS4-1-mut and all its combinatory mutants) severely disrupted the nucleolar targeting function (Fig. 5B and C). To further assess whether K356 and R359 within cluster 1 act as independent determinants, single-point mutants (N-NoLS4-356A and N-NoLS4-359A) were constructed (Fig. 5D). The results showed that a single mutation of either K356 or R359 drastically reduced the nucleolar localization rate to ~35%, which was nonetheless significantly higher than that of the complete cluster 1 mutant (16%) (Fig. 5E and F). These results indicate that the nucleolar localization activity of NoLS4 is highly dependent on the basic residues of cluster 1, with K356 and R359 acting synergistically as the core residues required for full NoLS4-mediated nucleolar localization.

### Disruption of NoLS4 abolishes nucleolar targeting of the N protein and impairs viral replication

**Fig 6:**
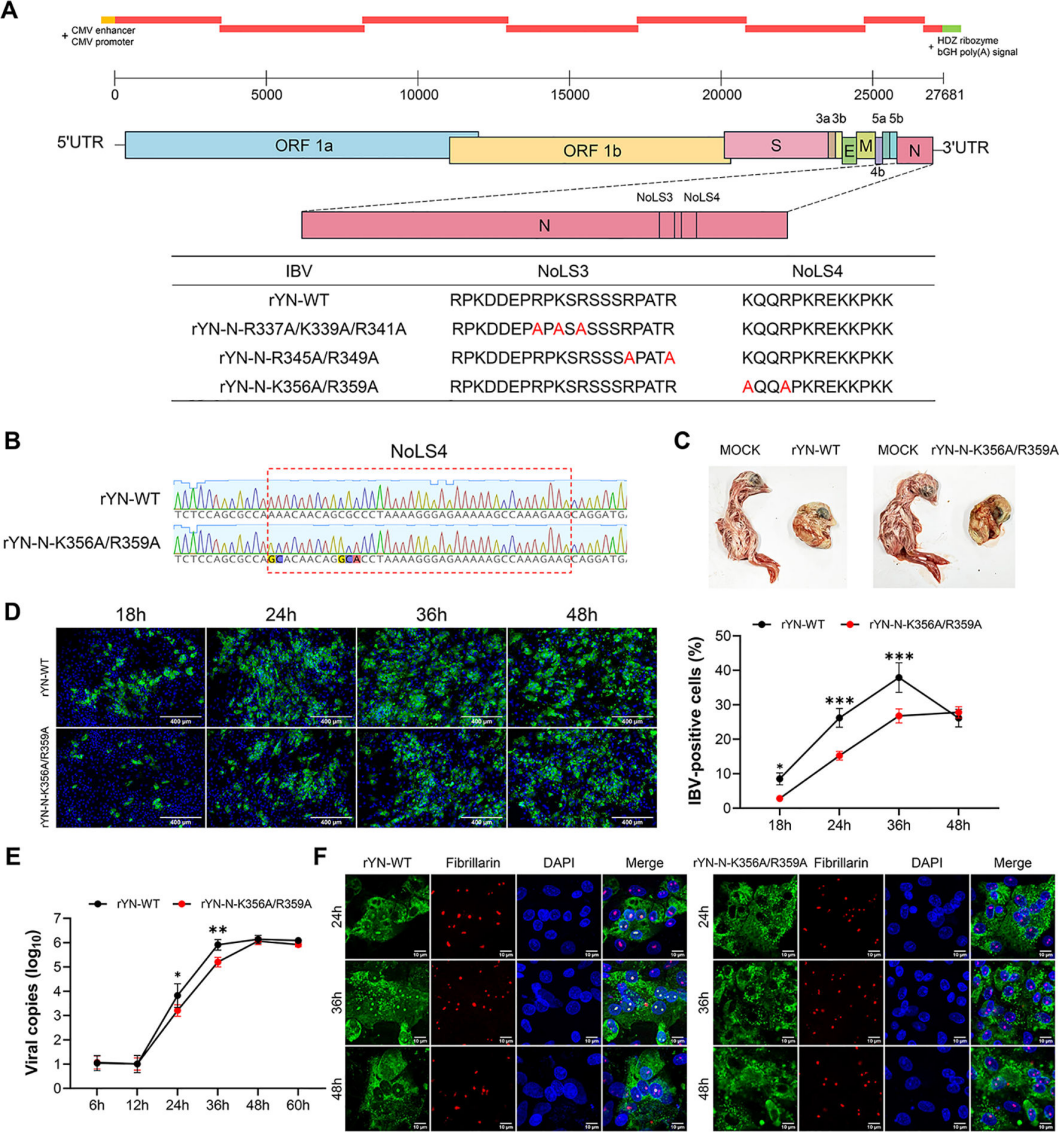
Disruption of NoLS4 abolishes nucleolar targeting of the N protein and impairs viral replication. (**A**) Schematic representation of the construction strategy for recombinant IBV mutants. Key alanine substitutions were introduced into the NoLS3 and NoLS4 regions of the N protein within the IBV genome using a reverse genetics platform (the introduced alanine mutations are indicated by red "A"s). (**B**) Sequence verification of the recombinant viruses. The N gene of the P5 recombinant viruses was verified by Sanger sequencing. The red dashed box highlights the nucleotide sequence of the NoLS4 region. (**C**) Assessment of viral infectivity in ECEs. Representative phenotypes of chicken embryos inoculated with PBS (MOCK), the wild-type recombinant virus (rYN-WT), or the mutant virus (rYN-N-K356A/R359A) are shown. (**D**) Assessment of viral replication in CEK cells by IFA. Left: CEK cells were infected with rYN-WT or rYN-N-K356A/R359A at an MOI of 0.01. Cells were fixed at 18, 24, 36, and 48 hpi and processed for IFA. The N protein is shown in green, and nuclei were counterstained with DAPI (blue). Scale bars: 400 μm. Right: Quantification of the percentage of IBV-positive cells at the indicated time points. Data are shown as mean ± SEM from three independent experiments (*n* = 3); >500 cells were scored in each experiment; *P < 0.05; ***P < 0.001 by two-way ANOVA followed by Bonferroni post hoc test. (**E**) Multi-step growth curves of the recombinant viruses. CEK cells were infected at an initial dose of 100 viral copies. Cell supernatants were harvested at various time points from 6 to 60 hpi, and the viral copy numbers were determined by RT-qPCR. (**F**) Subcellular localization of the N protein during viral infection. CEK cells were infected at an MOI of 0.04. At 24, 36, and 48 hpi, the cells were fixed and analyzed by IFA. N protein is shown in green, fibrillarin in red, and nuclei in blue. Scale bars: 10 μm. Data are shown as mean ± SEM from three independent experiments (*n* = 3); *P < 0.05; **P < 0.01 by two-way ANOVA followed by Bonferroni post hoc test.

To investigate the role of the N protein NoLS during viral infection, we used a TAR-based reverse genetics system to rescue wild-type and NoLS-mutant IBV strains (Fig. 6A). We successfully rescued the NoLS4 mutant strain rYN-N-K356A/R359A. In contrast, repeated attempts (three independent trials) to rescue the NoLS3 mutant strains (rYN-337A, 339A, 341A, and rYN-345A, 349A) were unsuccessful. Sanger sequencing of the N gene from the passage 5 (P5) rYN-N-K356A/R359A virus produced clear and single chromatogram peaks at the NoLS4 region, confirming the stable integration of the alanine substitutions into the viral genome (Fig. 6B). Morphological observation of the embryos post-inoculation revealed that chicken embryos inoculated with rYN-WT or rYN-N-K356A/R359A exhibited typical dwarfing and curling, which are characteristic of IBV infection, whereas embryos in the PBS mock-inoculated control group developed normally (Fig. 6C). We then assessed the effect of the NoLS4 substitutions on replication kinetics in CEK cells. IFA results at an MOI of 0.01 indicated that rYN-N-K356A/R359A exhibited fewer fluorescence-positive cells during the early-to-mid stages of infection (18–36 hpi) compared to rYN-WT (Fig. 6D). Consistent with these observations, viral growth-curve analysis revealed that the genome copy number of the mutant strain was significantly lower than that of the wild-type virus at 24 and 36 hpi during the logarithmic growth phase (Fig. 6E). To determine whether the NoLS substitutions altered N protein subcellular localization during authentic infection, CEK cells were infected with rYN-WT or rYN-N-K356A/R359A at an MOI of 0.04 and examined at 24, 36, and 48 hpi. In rYN-WT-infected cells, N localized to both the nucleolus and the cytoplasm, whereas in rYN-N-K356A/R359A infected cells, N localization was restricted to the cytoplasm, confirming that the altered localization observed in plasmid transfection experiments is recapitulated during virus infection (Fig. 6F). To rule out the possibility that differences in N protein expression levels might influence viral replication, we first compared the expression of wild-type and mutant N proteins in CEK cells. The results showed that the two proteins were expressed at comparable levels (Fig. S1A). We next assessed whether the mutation affected the intracellular stability of the N protein. At 24 h post-transfection, cycloheximide (CHX) was added to block *de novo* protein synthesis, and N protein degradation was monitored over time. The results showed that the mutant and wild-type N proteins exhibited similar degradation kinetics, indicating that this mutation had no appreciable effect on N protein stability (Fig. S1B). Given the central role of the N protein in viral RNA binding, we further examined whether this mutation altered its RNA-binding capacity using an RNA immunoprecipitation (RIP) assay. CEK cells were infected at an MOI of 0.01, and RIP was performed at 42 h post-infection. No significant difference in RNA-binding capacity was observed between the mutant and WT N proteins (Fig. S1C). These results demonstrate that disruption of the N protein NoLS (NoLS4) abolishes nucleolar targeting and substantially delays IBV replication in CEK cells.

## DISCUSSION

The subcellular localization of the N protein establishes the spatial framework required for its diverse functions. Although it is predominantly distributed in the cytoplasm to participate in viral replication and assembly, our study demonstrates that the IBV N protein also localizes to the nucleolus under both authentic viral infection and ectopic expression, corroborating early reports by Hiscox and colleagues (20). This nucleolar localization appears to be conserved among multiple coronavirus N proteins. Within the nucleolus, the N protein associates, directly or indirectly, with several nucleolar constituents, including nucleophosmin (NPM1/B23), fibrillarin (FBL), and nucleolin (NCL) (21, 22, 25, 31). These interactions perturb nucleolar architecture and alter the distribution and function of these proteins, resulting in the disruption of cell cycle progression. For instance, the PEDV N protein interacts with NPM1 and protects it from caspase-3-mediated cleavage, thereby prolonging host-cell survival and facilitating viral replication (22). Expression of the TGEV N protein induces cell cycle arrest at the G2/M phase and inhibits cytokinesis (32). Similarly, the SARS-CoV N protein delays S-phase progression in mammalian cell lines; this effect is proposed to slow host-cell proliferation and thereby allocate time and resources for viral genome replication and particle assembly (33). The conservation of these host-hijacking strategies indicates that nucleolar targeting of the IBV N protein is biologically significant and therefore warrants a detailed dissection of the molecular determinants that govern its nucleolar localization.

Using systematic truncation strategies, we precisely identified four highly conserved NoLSs within the IBV N protein. Consistent with our sequence analysis and previous reports, the most prominent feature of NoLSs is their enrichment in basic amino acid residues, particularly arginine and lysine (34). These positively charged residues typically cluster to create localized regions of high charge density. As a result, each NoLS displays a strong net positive charge and a high isoelectric point (pI), facilitating non-specific electrostatic interactions with negatively charged ribosomal RNA (rRNA) and acidic nucleolar proteins, such as NCL or B23 (35–37). This charge-driven retention mechanism underlies the accumulation of viral proteins in the nucleolus, in contrast to the importin-dependent nuclear import mediated by NLSs (38). Furthermore, unlike classical NLSs, NoLSs do not conform to a single, strictly conserved primary-sequence consensus and show substantial length variability, typically 7–30 amino acids in length (39). Notably, the majority of NoLSs are located within intrinsically disordered regions (IDRs) of viral proteins, which is in agreement with our finding that NoLS3 and NoLS4 map to the IDR3 segment (aa 330–409) of the IBV N protein (38, 40). The inherent structural flexibility of IDRs allows these motifs to undergo conformational adjustments, ensuring optimal adaptation to the complex molecular environment of the nucleolus (41).

Interestingly, NoLSs and NLSs often overlap both spatially and functionally. Numerous viral proteins—such as the Tat protein of human immunodeficiency virus type 1 (HIV-1), the assembly-activating protein (AAP) of adeno-associated virus type 2 (AAV2), and the ORF57 of herpesvirus saimiri (HVS)—contain basic amino acid-rich regions that mediate both nuclear import and nucleolar retention (26, 29, 42). In this study, NoLS3 (aa 330–349) and NoLS4 (aa 356–368) are embedded within a broader NLS (aa 330–368), conferring dual nuclear and nucleolar localization capabilities upon the N protein. However, IFA revealed that although the N protein is efficiently imported into the nucleus, it is predominantly sequestered in the nucleolus, with only a minor fraction distributed throughout the nucleoplasm. We propose that this distribution is likely modulated by an internal nuclear export signal (NES, ^291^LQLDGLHL^298^) within the N protein, which would promote nuclear export. These phenomena indicate that the subcellular distribution of the N protein is dynamic rather than static, reflecting a finely tuned spatial equilibrium governed by the interplay among NLS-mediated import, NoLS-mediated nucleolar retention, and NES-mediated export.

We found that NoLS3 (aa 330–349) and NoLS4 (aa 356–368), located at the C terminus, are essential for the nucleolar localization of the full-length N protein, and their cooperative action is required to efficiently retain the N protein in the nucleolus. This nucleolar localization mechanism, which involves the cooperation of multiple signals, is also common in other viral proteins. For example, nucleolar accumulation of influenza virus nucleoprotein (NP) depends on the combined action of multiple short sequence elements, a process proposed to facilitate efficient assembly of viral RNP complexes (43, 44). Similarly, bovine adenovirus type 3 (BAdV-3) pV contains several NLSs (aa 81–120, 190–210, and 380–389) and NoLSs (aa 21–50 and 380–389), with aa 380–389 functioning as both an NLS and a NoLS; these redundant, cooperative signals ensure efficient nucleolar entry of pV (45). While previous studies identified only a single NoLS (aa 71–78, substantially overlapping with our NoLS1), our findings indicate that this previously reported signal plays only a marginal role in the context of the full-length protein (28). Although NoLS1 and NoLS2 show nucleolar-targeting capacity as truncated fragments, their actual contribution is vastly overshadowed by the newly identified NoLS3 and NoLS4. This discrepancy is likely due to conformational masking in the intact protein, a hypothesis that warrants further experimental validation.

Fine-scale mutational analysis of the NoLSs in this study revealed a key feature of nucleolar targeting by the IBV N protein: the localization activities of NoLS3 and NoLS4 are not dictated by a single determinant residue but instead depend strongly on the cooperative action of clusters of basic residues and the overall positively charged microenvironment within each region. Specifically, NoLS3 requires the combined contributions of clusters 2 and 3, whereas NoLS4 depends on the joint action of K356 and R359 within N-terminal cluster 1. This mechanism of nucleolar localization is broadly conserved across numerous viral proteins. For instance, the Rev protein of human immunodeficiency virus type 1 (HIV-1) relies on multiple basic residues within its arginine-rich motif (ARM) to cooperatively mediate nucleolar targeting and RNA binding: mutation of a single arginine typically only reduces targeting efficiency, while deletion or substitution of large charged clusters markedly impairs nucleolar retention (46). Likewise, the C-terminus of AAV2 AAP contains up to five basic-residue clusters, and efficient nucleolar localization requires the combinatorial cooperation of at least four of these clusters (29). Such an evolutionary strategy confers mutational tolerance on the IBV N protein: during prolonged replication and natural evolution, substitutions at individual basic residues will not abolish nucleolar targeting provided that the region’s overall positive-charge environment is preserved.

We recently established a universal, rapid reverse genetics platform for IBV by systematically optimizing TAR-based technology (47). Using this platform, we successfully rescued a recombinant IBV strain (rYN-N-K356A/R359A) whose N protein lacked nucleolar localization capacity. Notably, this mutant exhibited reduced replication kinetics during the logarithmic growth phase of infection but ultimately reached a replication level comparable to that of the wild-type virus (rYN-WT). However, subsequent experiments demonstrated that the mutant protein exhibited expression levels, stability, and RNA-binding capacity comparable to those of the wild-type N protein. A plausible explanation is that disruption of NoLS4 impairs N-protein entry into the nucleolus, thereby weakening the virus’s ability to hijack specific nucleolar host factors and reducing replication efficiency during the early phase of infection. A similar pattern has been observed in other viruses: for instance, the bovine torovirus (BToV) N protein contains a highly overlapping NLS/NoLS. Recombinant BToV mutants that harbored NLS/NoLS mutations but retained nuclear accumulation capacity were successfully rescued, albeit with slightly reduced growth kinetics. In contrast, viruses that lost NLS/NoLS-mediated nuclear accumulation of the N protein failed to be rescued (48). Similarly, PRRSV carrying NoLS mutations in the N protein, after successful rescue, exhibited markedly reduced replication titers *in vitro* and strongly attenuated virulence *in vivo* (49). Notably, strains carrying mutations in NoLS3 could not be rescued, suggesting that the NoLS3 region may have functions beyond nucleolar targeting and may also participate in more essential processes during the viral life cycle.

Although our data indicate that nucleolar localization of the N protein is important for efficient IBV replication, the downstream consequences of this localization for host-cell biology remain unclear. Future investigations should clarify whether the disruption of nucleolar targeting affects cell-cycle progression, nucleolar stress signaling, nucleolar structure, and pre-rRNA biogenesis during infection. Further work is also warranted to determine the consequent impact on the localization and function of core nucleolar proteins, including nucleophosmin, nucleolin, and fibrillarin.

In summary, this study identified four NoLSs and an NLS within the N protein of IBV and demonstrated that the C-terminal NoLS3 and NoLS4 constitute the core determinants mediating its nucleolar localization. This targeting mechanism does not rely on any single amino acid residue but instead depends on the cooperative action of multiple clusters of basic residues. Importantly, efficient accumulation of the N protein in the nucleolus is essential for robust viral replication. These findings not only refine our understanding of the spatial localization mechanisms of coronavirus N proteins but also further underscore the pivotal role of the nucleolus in coronavirus infection.

## MATERIALS AND METHODS

### Animals

Specific-pathogen-free (SPF) embryonated chicken eggs (ECEs) were obtained from Beijing Boehringer Ingelheim Vital Biotechnology Co., Ltd. (Beijing, China).

### Viruses and cells

The IBV YN strain (GenBank JF893452.2) was maintained in our laboratory and propagated by inoculation into the allantoic cavity of 10-day-old SPF ECEs. Allantoic fluid was harvested 40 h post-inoculation, clarified by low-speed centrifugation (3,000 × *g*, 5 min), aliquoted, and stored at −80°C. Primary chicken embryo kidney (CEK) cells were isolated from 18-day-old SPF ECEs. Briefly, kidneys were aseptically excised, minced, washed with phosphate-buffered saline (PBS), and digested with 0.05% trypsin at 37°C for 20 min. The resulting cell suspension was filtered through a cell strainer, pelleted by centrifugation, and resuspended in complete culture medium. To enrich for epithelial cells, fibroblasts were depleted via differential adhesion. Following purification, the CEK cells were seeded into 24-well plates. CEK, Vero E6, and BHK-21 cells were cultured in Dulbecco’s modified Eagle’s medium (DMEM; Gibco, USA) supplemented with 10% fetal bovine serum (FBS) and 1% penicillin-streptomycin. All cell cultures were maintained at 37°C in a humidified incubator with 5% CO_2_.

### Plasmid construction and transfection

The full-length N gene was amplified from the cDNA of the IBV YN strain and cloned into the pCMV-Flag vector. The resulting pCMV-Flag-N plasmid served as the template for generation of a series of N gene truncations, which were subsequently subcloned into the pEGFP-C1 vector by Gibson assembly or by site-directed mutagenesis, where required. To construct the NoLS mutants, alanine-scanning mutagenesis targeting the basic amino acid residues within the NoLS regions was performed using the pCMV-Flag-N plasmid as the template. Primer sequences are provided in Table S1.

Vero E6 cells were seeded in 24-well plates and transfected at approximately 80% confluence. Immediately prior to transfection, the culture medium was replaced with antibiotic-free DMEM supplemented with 4% FBS. Transfection complexes were prepared by diluting 0.5 μg of plasmid DNA and 1 μL of Nulen PlusTrans reagent (Nulen, Shanghai, China) in Opti-MEM, gently mixing, and incubating at room temperature for 10 min. DNA–lipid complexes were then added dropwise to the wells; plates were gently swirled to ensure even distribution, and the cells were incubated in a humidified incubator at 37°C with 5% CO₂ for 24 h.

### Indirect immunofluorescence assay (IFA)

At the indicated time points post-transfection or post-infection, the cells were collected, fixed, and permeabilized in 200 μL of a 1:1 (vol/vol) methanol:acetone mixture at −20°C for 10 min, and subsequently blocked with blocking buffer (Beyotime Biotechnology, Beijing, China) for 15 min, unless otherwise stated. Cells were incubated with a monoclonal anti-IBV N protein antibody (HyTest, Finland; 1:2,000 dilution) and a monoclonal anti-fibrillarin antibody (Boster, Wuhan, China; 1:200 dilution) at 4°C for 12 h. Corresponding Alexa Fluor-conjugated secondary antibodies (Alexa Fluor 488-conjugated anti-mouse IgG [H + L] and Alexa Fluor 555-conjugated anti-rabbit IgG [H + L]; Cell Signaling Technology, USA; 1:500 dilution) were applied and incubated at 37°C for 1 h. Nuclei were counterstained with DAPI (Sigma-Aldrich, USA) at room temperature for 10 min. After three washes with PBST, coverslips were mounted with anti-fade mounting medium and imaged using an A1 HD25 confocal microscope (Nikon, Japan). Colocalization analysis was performed using Fiji ImageJ software. The Pearson correlation coefficient (PCC) was calculated to quantify the spatial overlap between the two fluorescent signals; PCC values range from −1 to +1, where + 1 indicates perfect positive correlation (complete colocalization), 0 indicates no correlation (random distribution), and −1 indicates perfect negative correlation.

### Conservation analysis of NoLS sequences

To determine whether the identified NoLSs are conserved across IBV strains, we retrieved the N protein sequences of 784 IBV isolates from the NCBI database. Sequences were subjected to multiple-sequence alignment using Geneious Prime (v2023.2.1; Biomatters Ltd, Auckland), with the results visualized as sequence logos using WebLogo (https://weblogo.threeplusone.com/create.cgi).

### Rescue of recombinant viruses

Recombinant viruses were rescued using a transformation-associated recombination (TAR)-based reverse genetics platform as described previously (47). In brief, using the pYES1L-rYN plasmid as template, eight contiguous genomic cDNA fragments (F1–F8) and one linearized vector fragment were amplified, with adjacent fragments designed to carry 40–60 bp overlapping homologous arms. Specifically, a CMV enhancer and CMV promoter were introduced at the 5′ end of fragment F1, and a hepatitis D virus (HDV) ribozyme together with the bovine growth hormone polyadenylation signal (bGH poly[A]) was fused to the 3′ end of fragment F8 to ensure precise transcription and processing of the viral genomic RNA in eukaryotic cells.

All fragments were co-transformed and assembled in strain Mav203 (Zomanbio, Beijing, China) using the lithium-acetate method. Recombinant plasmids recovered from assembled yeast clones were electroporated into Escherichia coli DH10B (Zomanbio, Beijing, China) for propagation, and plasmid DNA was extracted. Following whole-genome verification by Illumina deep sequencing, the verified plasmids together with the helper plasmid pCMV-Flag-N were transfected into BHK-21 cells to rescue the infectious virus.

Cell lysates harvested after three freeze–thaw cycles were inoculated into the allantoic cavity of 10-day-old SPF ECEs, and the allantoic fluid was collected 40 h post-inoculation. First-pass (P1) allantoic fluid was re-inoculated into 10-day-old SPF ECEs, and embryonic mortality and characteristic pathological changes were recorded at 144 h post-inoculation. After five consecutive passages, viral RNA was extracted, reverse-transcribed, and the N-gene region was amplified; Sanger sequencing confirmed the successful introduction of the intended mutation at the NoLS site. Primer sequences are listed in Table S1.

### Real-time quantitative PCR

Total RNA was extracted using the Total RNA Isolation Kit (Magen, Beijing, China) in accordance with the manufacturer’s instructions. The isolated RNA was then reverse-transcribed into complementary DNA (cDNA) using the StarScript III All-in-one RT Mix with gDNA Remover (Genstar, Beijing, China), thereby incorporating genomic DNA removal during the reverse-transcription step. Quantitative real-time PCR (qPCR) was subsequently performed on the resulting cDNA using the M5 HiPer Real-Time PCR Super Mix (Mei5bio, Beijing, China) following the manufacturer’s protocol.

### Growth kinetics evaluation of recombinant viruses

To evaluate the growth kinetics of the recombinant IBVs, primary CEK cells were inoculated with recombinant viruses at an input of 100 viral genome copies. After incubation for 1 h, the inoculum was removed, and the cells were washed three times with DMEM. At the indicated time points (6, 12, 24, 36, 48, and 60 h post-infection [hpi]), cell culture supernatants were collected, and viral genomic RNA was quantified by reverse transcription quantitative PCR (RT-qPCR). Amplification targeted the 5′ untranslated region (5′ UTR) of the IBV genome using the following primers: F, 5′-GTTGGGCTACGTTCTCGC-3′; R, 5′-CCTGTTTTAGGCTTGAAGCCAT-3′. Absolute quantification was derived from a standard curve according to the equation log_10_(viral RNA copies/μL) = −0.3141 × Ct + 11.314, where Ct represents the cycle threshold value.

### Western blotting

At the indicated time points after transfection, the culture supernatant was removed, and the cells were washed three times with PBS. Cells were lysed on ice for 15 min using radio-immunoprecipitation assay (RIPA) buffer with protease inhibitors, then centrifuged at 13,300 × *g* for 10 min. Supernatants were collected, mixed with loading buffer, and boiled for 10 min. Proteins were separated by SDS-PAGE and transferred to PVDF membranes. PVDF membranes were blocked with 5% skim milk for 2 h at room temperature, washed three times with TBST, and then incubated overnight at 4°C with anti-GAPDH monoclonal antibodies (Proteintech, Wuhan, China; 1:10,000 dilution) or anti-IBV nucleocapsid (N) polyclonal antibody (laboratory-made; 1:4,000 dilution). After three TBST washes, the membranes were incubated with HRP-conjugated goat anti-rabbit IgG secondary antibodies (Bioss, Beijing, China, 1:10,000 dilution) for 1 h at room temperature. ECL substrate (Beyotime Biotechnology, Shanghai, China) was added, and membranes were imaged using a ChemiDoc MP system (Bio-Rad, California, USA).

### RNA immunoprecipitation (RIP)

To assess the binding ability of the N protein to viral genomic RNA, CEK cells were infected with recombinant rYN-WT or rYN-N-K356A/R359A at an MOI of 0.01. At 42 hpi, the cells were harvested and lysed in western and IP Cell Lysis Buffer (Beyotime Biotechnology, China). After clarification, an aliquot of each lysate was retained as the input control, and the remaining lysate was subjected to immunoprecipitation with an anti-N antibody. The resulting immune complexes were captured with Protein A/G PLUS-Agarose (Santa Cruz Biotechnology, USA), thereby generating agarose bead-bound N–RNA complexes. After extensive washing to remove nonspecifically associated materials, RNA was extracted from the immunoprecipitated complexes and analyzed by RT-qPCR to quantify the abundance of viral genomic RNA in the input and immunoprecipitated fractions. In parallel, the levels of N protein in the input and immunoprecipitated samples were assessed by western blotting.

### Statistical analysis

Statistical analyses were performed using GraphPad Prism version 10.0 (GraphPad Software Inc., San Diego, CA, USA). Comparisons involving a single categorical factor with more than two levels were analyzed using one-way ANOVA, followed by Dunnett post hoc test, whereas analyses involving two independent variables were performed using two-way ANOVA, followed by Bonferroni post hoc test, as specified in the figure legends. In all cases, *P  <  0.05; **P  <  0.01; ***P  <  0.001; ns, not significant *P*  >  0.05. All experiments subjected to statistical testing were independently repeated at least three times to ensure reproducibility.

## Data Availability

All data supporting the findings of this study are provided within the article and its supplemental material.

## SUPPLEMENTAL MATERIAL

Supplemental material jvi.00433-26-s0001.docx Table S1; Fig. S1.

ASM does not own the copyrights to Supplemental Material that may be linked to, or accessed through, an article. The authors have granted ASM a non-exclusive, world-wide license to publish the Supplemental Material files. Please contact the corresponding author directly for reuse.
