# Supplementary material for: Characterization of nucleolar localization signals in the avian infectious bronchitis virus nucleocapsid protein and their critical role in viral replication
Source: J Virol. 2026 Jun 4;100(6):e00433-26. doi: 10.1128/jvi.00433-26 (PMC13288618; doi:10.1128/jvi.00433-26)
Supplement: Supplemental material — Table S1; Fig. S1. [file jvi.00433-26-s0001.docx]

**Supplementary Material**

**Table S1.** Primers used in the study.

| **Primer Name** | **Sequences (5’-3’)** | **Purpose** |
| --- | --- | --- |
| N-F | *acaaggacgacgatgacaag*ATGGCAAGCAGTAAGGCATTTGGA | N gene amplification |
| N-R | *cgcggccgcggtacctcgag*TCAAAGTTCATTTTCACCAAGTGCTG |  |
| E-N-F | *gcatggacgagctgtacaag*GGAGGCGGGGGTAGTGCAAGCAGTAAGGCATTTGGA | Amplification of the N protein gene and its truncated variants for fusion with the EGFP tag. |
| E-N-R | *gatcccgggcccgcggtacc*TCAAAGTTCATTTTCACCAAGTGCTG |  |
| N-72-85-F | *acgccaagccaggtataagc*CGGGCAAAGGTGGAAGAAAATAACCCGGGATCCACCGGATCTA |  |
| N-72-85-R | *gcttatacctggcttggcgt*CTACTACCCCCGCCTCCCGCGTACAGCTCGTCCATGCCGA |  |
| 1-71-F | *tcggcatggacgagctgtac*GCGGGAGGCGGGGGTAGTATGGCAAGCAGTAAGGCATTTGG |  |
| 1-71-R | *ccagtacccatgctgctggc* |  |
| 86-156-F | *gccagcagcatgggtactgg*CCAGTCCCAGATGCGTGGT |  |
| 86-156-R | *gatcccgggcccgcggtacc*TTAGTCCCAACGGAAATTTCCATCAGG |  |
| N-1-156-F | *tcggcatggacgagctgtac*GCGGGAGGCGGGGGTAGTATGGCAAGCAGTAAGGCATTTGG |  |
| N-1-156-R | *gatcccgggcccgcggtacc*TTAGTCCCAACGGAAATTTCCATCAGG |  |
| N-157-218-F | *tcggcatggacgagctgtac*GCGGGAGGCGGGGGTAGTTTTATTCCTCTGAATCGTGGTAGGAGTG |  |
| N-157-218-R | *tatctagatccggtggatcc*TTACTTAGCCTTTGTAATGCGGGAAC |  |
| N-162-167-F | *ggggtagtcgtggtaggagt*GGAAGGTAACCCGGGATCCACCGGATCT |  |
| N-162-167-R | *actcctaccacgactacccc*CGCCTCCCGCGTACAGCTCGTCCATGCCG |  |
| N-177-188-F | *ctagggcaccttcaagagaa*GGTTCTCGTGGAAGATAACCCGGGATCCACCGGATCTAG |  |
| N-177-188-R | *ttctcttgaaggtgccctag*AACTACCCCCGCCTCCCGCGTACAGCTCGTCCATGCCG |  |
| N-199-218-F | *ttcaagaccagcagaaaaag*GGTTCCCGCATTACAAAGGCTAAGTAACCCGGGATCCACCGGATCTAG |  |
| NoLS1-F | *acaagccgcatatgcacc*GGGCGCAGGTGGAGCAGCACCAGTCCCAGATGCGTGGT | Alanine substitution mutagenesis of basic amino acid residues within the NoLS motif. |
| NoLS1-R | *ggtgcatatgcggcttgt*GCTGCCCAGTACCCATGCTGCTGGC |  |
| NoLS2-F | *cagcaggcagcaggttccg*CAATTACAGCAGCTGCAGCAGATGAGATGATTCATCGCCG |  |
| NoLS2-R | *cggaacctgctgcctgctg*GTCTTGAATAATTGCTGCCGCTGCAGCAATAAGATTTTCTTCAGCCCCAC |  |
| NoLS3-F | *cagcaccagcatcagcat*CAAGTTCAGCACCTGCTACAGCAGGAAGTTCTCCAGCGCCA |  |
| NoLS3-R | *atgctgatgctggtgctg*GTTCATCATCTGCTGGTGCTGTTCCTACACCATCAACGCAC |  |
| NoLS4-F | *agcagaggcagcaccagc*AGCACAGGATGATGAAGTGGATAAAGCATTGAC |  |
| NoLS4-R | *gctggtgctgcctctgct*GCAGGTGCCTGTTGTGCTGGCGCTGGAGAACTTCCT |  |
| 3-1-F | *gcaccagcagatgatgaacc*AAGACCAAAATCACGCT |  |
| 3-1-R | *ggttcatcatctgctggtgc*TGTTCCTACACCATCAACGCACT |  |
| 3-2-F | *accagcaccagcatcagcat*CAAGTTCAAGACCTGCTACAAGAGGA |  |
| 3-2-R | *atgctgatgctggtgctggt*TCATCATCCTTTGGACGTGT |  |
| 3-3-F | *caagttcagcacctgctaca*GCAGGAAGTTCTCCAGCGCCAAAAC |  |
| 3-3-R | *tgtagcaggtgctgaacttg*AGCGTGATTTTGGTCT |  |
| 3-4-F | *tgatgaaccagcaccagcat*CAGCATCAAGTTCAAGACCTGCTACAAGAG |  |
| 3-4-R | *atgctggtgctggttcatca*TCTGCTGGTGCTGTTCCTACACCATCAACGCACTG |  |
| 3-5-F | *accaaaatcacgctcaagtt*CAGCACCTGCAACAGCAGGAAGTTCTCCAGCGCCAAAAC |  |
| 3-5-R | *aacttgagcgtgattttggt*CTTGGTTCATCATCTGCTGGTGCTGTTCCTACACCATCAACGCAC |  |
| 3-6-F | *cagcatcagcatcaagttca*GCACCTGCTACAGCAGGAAGTTCTCCAGCGCCAAAAC |  |
| 3-6-R | *tgaacttgatgctgatgctg*GTGCTGGTTCATCATCCTTTGGACGTG |  |
| 337A-F | *aaaggatgatgaaccagcacc*AAAATCACGCTCAAGTTCAAGACC |  |
| 337A-R | *ggtgctggttcatcatccttt*GGACGTGT |  |
| 339A-F | *agaccagcatcacgctcaag*TTCAAGACCTG |  |
| 339A-R | *cttgagcgtgatgctggtct*TGGTTCATCATCCTTTGGAC |  |
| 341A-F | *ccaaaatcagcatcaagttc*AAGACCTGCTACAAGAGG |  |
| 341A-R | *gaacttgatgctgattttgg*TCTTGGTTCATCATCCT |  |
| 345A-F | *ctcaagttcagcacctgcta*CAAGAGGAAGTTCTCCAG |  |
| 345A-R | *tagcaggtgctgaacttgag*CGTGATTTTGGTCT |  |
| 349A-F | *agacctgctacagcaggaag*TTCTCCAGCGCCAAAAC |  |
| 349A-R | *cttcctgctgtagcaggtct*TGAACTTGAGCG |  |
| 4-1-F | *gcacaacaggcacctaaaag*GGAGAAAAAGCCAAAGAAG |  |
| 4-1-R | *cttttaggtgcctgttgtgc*TGGCGCTGGAGAACTTCCTC |  |
| 4-2-F | *cagcgccctgcagcagaggc*AGCACCAAAGAAGCAGGATGATGAAGTGGA |  |
| 4-2-R | *gcctctgctgcagggcgctg*TTGTTTTGGC |  |
| 4-3-F | *gcagcacaggatgatgaagt*GGATAAAGCATTG |  |
| 4-3-R | *acttcatcatcctgtgctgc*TGGCTTTTTCTCCCTTTTAGGGC |  |
| 4-4-F | *gcacctgcagcagaggcagc*ACCAAAGAAGCAGGATGATGAAGTGGAT |  |
| 4-4-R | *gctgcctctgctgcaggtgc*CTGTTGTGCTGGCGCTGGAGAACTTCCT |  |
| 4-5-F | *cctaaaagggagaaaaagcc*AGCAGCACAGGATGATGAAGTGGATAAAGCATTGAC |  |
| 4-5-R | *ggctttttctcccttttagg*TGCCTGTTGTGCTGGCGCTGGAGAACTTCCTC |  |
| 4-6-F | *tgcagcagaggcagcaccagc*AGCACAGGATGATGAAGTGGATAAAGCATTGACC |  |
| 4-6-R | *gctggtgctgcctctgctgca*GGGCGCTGTTGTTTTGGC |  |
| 356A-F | *tctccagcgccagcacaaca*GCGCCCTAAAAGGGAGAAA |  |
| 356A-R | *tgttgtgctggcgctggaga*ACTTCCTC |  |
| 359A-F | *ccaaaacaacaggcacctaa*AAGGGAGAAAAAGCCAAAGAAG |  |
| 359A-R | *ttaggtgcctgttgttttgg*CGCTGGAGAA |  |
| F1-F | ATATAGAACCCGAACGACCGAGC | Rescue of Recombinant Strains |
| F1-R | TCCTGCTCTACTTGTGGCTCCTC |  |
| F2-F | GTTCCTACAAAAGAAGTTGTGCCTCA |  |
| F2-R | TATTCATCTCCATATGATAGTAGCCCAAAC |  |
| F3-F | TTACATTGTGTTGTTTGGGCTACTATCAT |  |
| F3-R | CACATCCATTAGCAAGGGGTATCAG |  |
| F4-F | GTAGCAGTGAGGCTCGGCTGAT |  |
| F4-R | GTGAAAGTGAACCATTAAGTAAAGTACGTGG |  |
| F5-F | TGATCCTGCACAATTGCCAGCAC |  |
| F5-R | GCACTACATAGTGCAAACAAAACAGTCAC |  |
| F6-F | TAGAGATGTTGGGGAAGTCACTGTTAATAG |  |
| F6-R | GCGGGAGTTTGAACCATTAAACAGAC |  |
| F7-3-2-F | AATGATGTGGTAACTTAACAATACAGACC |  |
| F7-3-2-R | TGTAGCAGGTCTTGAACTTGATGCTGATGCTGGTGCTGGTTCATCATCCTTTGGACGTG |  |
| F8-3-2-F | GATGAACCAGCACCAGCATCAGCATCAAGTTCAAGACCTGCTACAAGAGG |  |
| F8-3-2-R | CCGTTATAGTTACGCGCTTCCTCG |  |
| F7-3-3-F | AATGATGTGGTAACTTAACAATACAGACC |  |
| F7-3-3-R | TTGGCGCTGGAGAACTTCCTGCTGTAGCAGGTGCTGAACTTGAGCGTGATTTTGGTCTT |  |
| F8-3-3-F | GCTCAAGTTCAGCACCTGCTACAGCAGGAAGTTCTCCAGCGCCAAAAC |  |
| F8-3-3-R | CCGTTATAGTTACGCGCTTCCTCG |  |
| F7-4-1-F | AATGATGTGGTAACTTAACAATACAGACC |  |
| F7-4-1-R | TTTCTCCCTTTTAGGTGCCTGTTGTGCTGGCGCTGGAGAACTTCCTCTT |  |
| F8-4-1-F | GAGGAAGTTCTCCAGCGCCAGCACAACAGGCACCTAAAAGGGAGAAAAAGCCAAAGAAG |  |
| F8-4-1-R | CCGTTATAGTTACGCGCTTCCTCG |  |
| pYES1L-F | CCTCGCCGCAGTTAATTAAAGTCAGTG |  |
| pYES1L-R | CGGCGGTATCAGCGCGGCCG |  |
| N-CE-F | GACACCTAAGCTTCAACCTGATGG | Identification of NoLS mutation in the N gene of recombinant strains |
| N-CE-R | TCAAAGTTCATTTTCACCAAGTGCTG |  |

F: forward primer; R: reverse primer. Homology arms are indicated in lowercase italics.

**
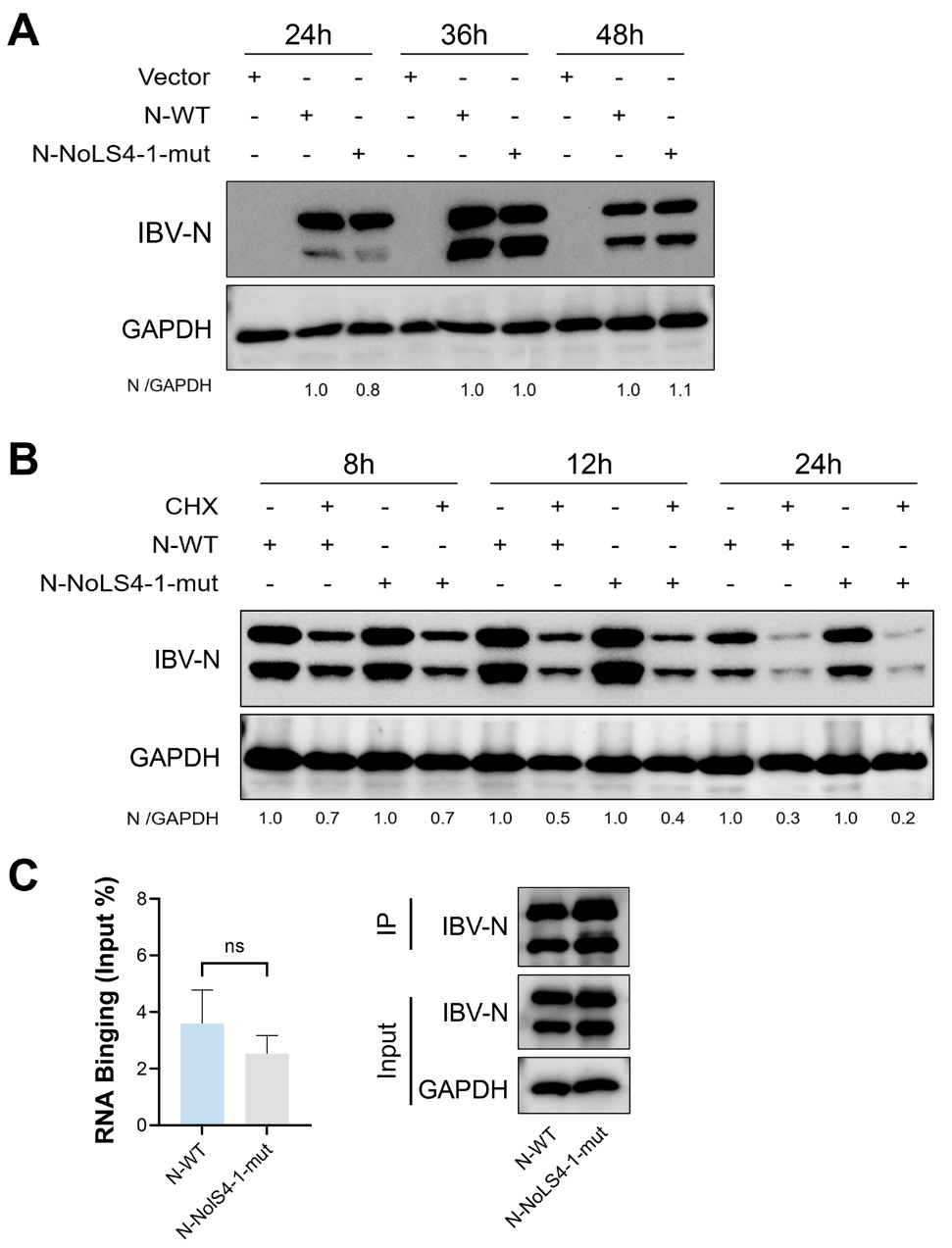
**

**Fig S1** The mutation does not affect N protein expression, stability, or RNA-binding capacity. (A) CEK cells were transfected with plasmids expressing N-WT or N-NoLS4-1-mut, and N protein expression was assessed at the indicated time points. (B) At 24 h after transfection with plasmids expressing N-WT or N-NoLS4-1-mut, CHX (50 μM final concentration) was added to block de novo protein synthesis, and N protein levels were analyzed by Western blotting. GAPDH was used as a loading control. (C) CEK cells were infected with recombinant rYN-WT or rYN-N-K356A/R359A virus at an MOI of 0.01, and RIP assays were performed at 42 hpi. The RNA recovery efficiency (input%) of mutant and wild-type N proteins was quantified by RT-qPCR as a percentage of input, and cell lysates were analyzed by Western blotting. Data are shown as mean ± SEM from 3 independent experiments (n = 3); ns, not significant by Student’s t-test.
